# Mixed feelings: general practitioners’ attitudes towards eHealth for stress urinary incontinence - a qualitative study

**DOI:** 10.1186/s12875-019-0907-x

**Published:** 2019-01-26

**Authors:** Lotte Firet, Chrissy de Bree, Carmen M. Verhoeks, Doreth A. M. Teunissen, Antoine L. M. Lagro-Janssen

**Affiliations:** Department of Primary and Community Care, Unit Gender in Primary and Transmural Care, Radboud university medical center, Geert Grooteplein 21, 6500 HB Nijmegen, the Netherlands

**Keywords:** eHealth, Stress urinary incontinence, General practitioners, Attitude of health personnel, Qualitative research, Self-management

## Abstract

**Background:**

Stress urinary incontinence (SUI) is the most prevalent subtype of urinary incontinence and is a bothering condition in women. Only a minority of those with SUI consult a general practitioner (GP). EHealth with pelvic floor muscle training (PFMT) is effective in reducing incontinence symptoms and might increase access to care. The role of the GP regarding such an eHealth intervention is unknown. The aim of the study is to gain insight into the attitudes towards a PFMT internet-based, eHealth, intervention for SUI.

**Methods:**

A qualitative study was conducted. Data were collected through semi-structured interviews among purposively sampled GPs. Audio records were fully transcribed, and analysed thematically.

**Results:**

Thirteen GPs were interviewed, nine females and four males. Three themes emerged: appraisal of eHealth as a welcome new tool, mixed feelings about a supportive role, and eHealth is no cure-all. GPs welcomed eHealth for SUI as matching their preferences for PFMT and having advantages for patients. With eHealth as stand-alone therapy GPs were concerned about the lack of feedback, and the loss of motivation to adhere to the intervention. Therefore, GPs considered personal support important. The GP’s decision to recommend eHealth was strongly influenced by a woman’s motivation and her age. GPs’ treatment preferences for elderly are different from those for young women with SUI; both PFMT and eHealth are perceived less suitable for older women.

**Conclusion:**

EHealth with PFMT fits into the GPs’ routine practice of SUI and adds value to it. Although there is evidence that eHealth as a stand-alone intervention is effective, GPs consider personal support important to supplement the perceived shortcomings. Probably GPs are not aware of, or convinced of the existing evidence. Training should address this issue and should also focus on common misunderstandings about regular care for women with SUI, such as the idea that PFMT is not suitable for the elderly. Improving GPs’ knowledge that eHealth can be a stand-alone therapy for SUI facilitates the implementation in daily care.

**Electronic supplementary material:**

The online version of this article (10.1186/s12875-019-0907-x) contains supplementary material, which is available to authorized users.

## Background

Urinary incontinence is a common condition in women and has a major impact on quality of life. Stress urinary incontinence (SUI) is the most prevalent subtype of incontinence and is defined as the complaint of involuntary urinary leakage on effort or exertion, or on sneezing or coughing [1–3]. The overall prevalence rate of urinary incontinence varies between 25 and 48%, according to two European studies [1, 3]. Among women who report having urinary incontinence, the prevalence of SUI varies between 21 and 33% with a peak prevalence between 40 and 49 years [1, 3]. Although urinary incontinence is not a life-threatening disease, it has major consequences in daily life. Women report the fear of being smelt; further, they feel embarrassed and have a low self-esteem [4]. Commonly women experience being limited in social or physical activities [5].

Among women with SUI only one in three seek professional help [6, 7]. As for women with other types of urinary incontinence, barriers for seeking help are shame, a lack of knowledge about treatment options, and the idea that incontinence is a consequence of giving birth or ageing [8, 9]. Women with severe symptoms or an impaired quality of life, are more likely to consult a healthcare worker [5-7]. Once women seek help for urinary incontinence, they mostly consult a general practitioner (GP) [6].

GPs encounter difficulties in providing the treatment to women with SUI that is recommended in guidelines [10]. The recommended first-line treatment for SUI consists of lifestyle advice, pelvic floor muscle training (PFMT), or pessary placement [11, 12]. GPs perform the diagnostic procedure according to available guidelines [10], but only one-third of women receive optimal treatment [6]. Factors that prevent GPs from providing optimal treatment are a lack of time, a lack of knowledge, and the feeling of not being capable to coach PFMT [10, 13, 14]. Although GPs experience barriers in providing care for urinary incontinence, they consider optimising treatment of SUI important [15].

A possible solution to improve care for SUI is internet-based therapy, eHealth. EHealth interventions have shown to be effective for various health-related topics for example, substance abuse, mental health, and chronic pain [16]. For SUI, there is also evidence that an eHealth intervention is effective. Two Swedish randomised controlled trials (one with an internet-based and one with a mobile phone intervention) and one observational feasibility study (internet-based intervention) have been conducted in this area [17–19]. The Swedish trials showed that symptom severity and incontinence-related quality of life improved significantly after women received an Internet-based intervention, or a mobile phone app intervention with PFMT [17, 18]. During the Internet-based intervention, there was no face-to-face contact, but a urotherapist sent reminders via e-mail and was available for questions if needed. Most women appreciated this contact because it motivated them, and they felt supported without being exposed [20]. The results of these studies cannot be simply added up as they differed in the application they used; internet-based or mobile-phone.

The role of GPs regarding an eHealth intervention for SUI is unknown. Possibly, women consult their GP with questions about an eHealth intervention after they started the intervention without having consulted a GP. Alternatively, GPs might recommend women with SUI to use eHealth, which could be attractive especially for those GPs with difficulties in providing regular care for SUI. Thus, the GP could become involved when eHealth for SUI would be implemented within the health care system. To implement an eHealth intervention successfully, the stakeholders’ attitudes need to be explored [21, 22]. Therefore this study aims to get insight into the GPs’ attitudes towards an internet-based, eHealth, intervention for women with SUI.

## Method

### Aim

Our study aims to gain insight into the GPs’ attitudes towards an eHealth intervention for SUI.

#### Study design

Within this qualitative study semi-structured interviews were carried out among GPs to create in-depth insight into the GPs’ attitudes towards eHealth to treat SUI.

#### Participants

Through purposive sampling, GPs were recruited by ten GPs of the Dutch College of General Practitioners (NHG) expert group on urogynaecological diseases (ugynHAG), whose practice locations were distributed throughout the Netherlands. Each GP from this expert group was asked to approach two GPs from their region who did not belong to the urogynaecology expertise group. We strived to include GPs with a broad variation in background characteristics to guarantee a wide range of views. The specialised GPs sent the e-mail addresses of participating GPs to the researcher. The researcher contacted the GPs by e-mail and provided information about the study through a letter. GPs were informed that there is evidence that eHealth could be an effective treatment modality for women with SUI [18]. They were also told that the eHealth application was defined as an eight-step internet-based training with PFMT and information about different aspects of SUI. The GPs who agreed to participate gave their written consent and thereafter the interviews took place.

#### Data collection

In November 2016, the interviews were conducted by a trained medical student (CB). The interview guide was based on literature, and on the expertise of the supervising committee. The interview guide was adjusted after two pilot interviews (Additional file 1). Key topics were experiences with routine practice for SUI and attitudes towards an eHealth intervention for SUI based on PFMT. We explicitly questioned opinions about support because literature showed that women thought personal support would increase their adherence to an eHealth treatment [23]. Interviews lasted 30 min on average, and they were held either face-to-face or by telephone, based on the individual GP’s preference. The interviewer and the GP did not know each other before the study started.

#### Analysis

Interviews were audio recorded and fully transcribed verbatim anonymously. Transcripts were not returned to the participants for comment. After every three interviews, two researchers (CV, CB) independently analysed the transcripts by using thematic coding through the qualitative software programme Atlas.ti, version 7.1.5. They met regularly to discuss their codes until consensus was reached on all codes. In case of disagreement, two other researchers (DT, LF) gave their opinions after reading the transcripts. Data saturation was reached after 11 interviews, as no new codes emerged. The last two interviews have been conducted because the appointments for interviews were already set, and indeed they revealed no new findings. All codes were clustered into categories, and during sessions with the research committee (all authors), three final themes emerged. No member checking occurred after data analysis. The consolidated criteria for reporting qualitative research (COREQ-criteria) [24] were applied to this manuscript (Additional file 2). To illustrate the main results, quotes are displayed with identifier number, sex and age category. Quotes were translated by a native English speaker. The words *few, several, many, most*, or *all* indicated that 1–3, 4–6, 7–9, 10–12, or 13 participants respectively shared an opinion.

#### Ethical and safety issues

This study was approved as part of a broader project on eHealth for stress urinary incontinence (file number 2016–2721). The research ethics committee of the Radboud university medical center, Nijmegen, replied positive on this request in November 2016.

## Results

Thirteen GPs participated in this study; nine of these were female (Table 1). Eight GPs were younger than 45 years old. Most GPs had their practice in an urban area. As reasons for non-participation, GPs mentioned practical aspects, such as limited time. Three themes emerged from data analysis: appraisal of eHealth as a welcome new tool, mixed feelings about a supportive role, and eHealth is no cure-all.

**Table 1:** Demographic characteristics of participants

Characteristics of 13 participating GPs	*n* (%)
Sex	
Female	9 (69%)
Male	4 (31%)
Age	
≤ 45 years	8 (62%)
> 45 years	5 (38%)
Practice type	
Group	11 (85%)
Health centre	2 (15%)
Practice location	
Urban	12 (92%)
Rural	1 (8%)
Distribution within the Netherlands	
North Netherlands	0 (0%)
East Netherlands	5 (38%)
South Netherlands	4 (31%)
West Netherlands	4 (31%)

### Appraisal of eHealth as a welcome new tool

PFMT was preferred by all GPs because it is effective, evidence-based, non-invasive, and non-pharmaceutical. Most GPs referred patients with SUI to a specialised pelvic floor physiotherapist for PFMT. Several GPs also provided information via leaflets, or they recommended their patients to use information on https://www.thuisarts.nl/ [25], a certified website of the Dutch College of General Practitioners. Referral for invasive surgical therapy was mentioned as a last resort option only for specific patients.

*“[ PFMT is preferred] because you can act on your complaints yourself without taking medication. And in the end, it is effective if women know how to use their pelvic floor muscle.” (GP6, female, 37).*

EHealth matched with the GP’s first-choice treatment for women with SUI because the eHealth programme was based completely on PFMT. Most GPs mentioned approachability and flexibility as advantages of eHealth that could increase access to care for women who would otherwise not seek help because of time restriction, shame, or resistance to physical examination. Women could perform exercises in their own time and pace without interference of a healthcare provider. Another advantage of eHealth was that women can start therapy without a diagnostic procedure of a GP. A few GPs acknowledged that they had a blind spot to detect SUI, which lowered the chance of women to be accurately treated.

*“Patients do not have the fuss anymore to undress themselves for a physical examination and physiotherapy. I think everybody wants to solve this alone at home, regardless of taboos.” (GP7, female, > 45 y)*

*“I think we only see the tip of the iceberg, and that we as doctors do not ask enough about those symptoms.” (GP8, male, ≤ 45 y).*

Furthermore, many GPs perceived eHealth as financially attractive both for patients and for society, as some women were not fully reimbursed for physiotherapy by standard health insurance. One GP warned against eHealth being used as an economic replacement for any other treatment.

“*It should be avoided that health insurance companies think: well, we have got eHealth, now we do not have to pay for other treatments anymore.” (GP4, male, > 45 y).*

For GPs, the advantages of eHealth for their own daily practice were also mentioned. Several GPs welcomed the existence of a new tool: they felt they had something extra to offer. EHealth could also save consultation time, as GPs had neither to explain PFMT nor to write referral letters. One GP, a frequent user of eHealth for mental health problems, emphasised that the logical set up of information by eHealth structured his consultations with patients. Before GPs would recommend an eHealth intervention, they emphasised a need for an evidence-based and accessible intervention without financial consequences. To enhance access, a few GPs suggested to integrate eHealth within frequently visited web pages, such as https://www.thuisarts.nl/ [25].

*“It is always good to add a new tool to your assortment of treatment options.”(GP7, female, > 45 y).*

*“If you are going to implement eHealth into our practices, you will encounter resistance when we have to install the eHealth application ourselves [...] that is the advantage of thuisarts.nl, which you can recommend to patients [...] keep the doctor lazy.” (GP13, female, ≤ 45 y).*

### Mixed feelings about a supportive role

GPs had mixed feelings about personal support during an eHealth intervention because on the one hand they preferred eHealth as self-sufficient tool, but on the other hand they were concerned that currently the effect of the training could not do without personal support. They emphasised that the effect of PFMT, either with eHealth or with regular care, depended on providing support because women commonly lack motivation to adhere to PFMT. Also feedback on the way women contracted their pelvic floor muscles was considered important. One GP thought that women who were unaware of their pelvic floor muscle were not suitable for eHealth. Women who performed the exercises incorrectly might not achieve any effect of PFMT, and several GPs were concerned that these women became discouraged to seek further help.

*“There are some people who stop feeling things beneath their diaphragm, so they have no clue of what happens down there. Of course, those people are not suitable for this eHealth.” (GP4, male, > 45 y).*

The opinions about how and by whom support should be provided varied among GPs. Several GPs suggested that less intensive support could take place within primary care. Several weeks after a woman started with eHealth, either the GP or the nurse practitioner could ask a woman whether she noticed improvement of SUI. This type of support is in line with their routine practice because GPs highly appreciated following their patient’s progress for example by updates from the physiotherapist about the results of PFMT. The idea to collaborate with nurse practitioners was based on GPs’ experiences with eHealth for mental health problems. A few GPs mentioned that these nurses had more time to motivate women. One GP considered it as her own moral duty to strongly motivate women to adhere to PFMT.

*“People who accept their urinary incontinence needed to be activated [...] it is a social choice [...] to reduce the amount of pads.” (GP11, female, > 45 y).*

Several GPs thought more profound support had to be performed by the physiotherapist, and therefore eHealth should not be implemented within general practice. A few GPs felt incapable of assessing the strength of the pelvic floor muscle, which restricted them in monitoring the effect of PFMT. They acknowledged having less affinity with SUI or pelvic floor problems.

A few GPs were concerned that, without their interference, women would start eHealth without having a correct diagnosis. GPs thought that it is their role to diagnose SUI and to rule out physical abnormalities such as an urine tract infection or a prolapse.

*“EHealth is suitable only for specific patients if the diagnosis is correct. Therefore, patients with an overflow bladder should not use eHealth. Thus, the indication assessment has to be right before recommending eHealth.” (GP9, female, > 45 y).*

### EHealth is no cure-all

GPs emphasised that eHealth with PFMT is not a solution for all women with SUI. They mentioned that women who lack motivation for PFMT will not be helped by eHealth either. For example, a woman who already performed regular PFMT without success would not be motivated to start with eHealth. Several GPs also explicitly mentioned that a woman’s preference for a specific treatment influenced their decision to recommend eHealth.

*“Whether I recommend eHealth depends on the patient. Some women would prefer personal support by a specialised pelvic floor muscle physiotherapist […] but when they have no specific preference, I would suggest to start with eHealth.” (GP13, female, ≤ 45 y).*

Beside motivation, GPs named other factors that were required before they would recommend eHealth. Women with visual impairment, lower education, and a language barrier would be less suitable for eHealth. Furthermore, GPs highlighted that patients with severe incontinence, psychiatric diseases, complex co-morbidity, or a history of sexual abuse might need more help than eHealth could offer.

Age was also named by many GPs as a determinant factor in care for women with SUI. Younger women were perceived to benefit more from eHealth because time- consuming jobs and/or the time needed to spend on care for children restricted them in visiting a physiotherapist. A few GPs thought that eHealth would perfectly suit post-partum women because, during pregnancy gymnastics, they might already have gained skills on how to contract their pelvic floor muscles. In routine practice several GPs were more inclined to refer a young women to the physiotherapist or to the specialist. For younger women, SUI was considered unacceptable and more bothersome, partly because they might be more sexually active. A few GPs thought that incontinence in young women could have been caused by an underlying disease that needed to be ruled out by a specialist.

*“I think the younger population, especially those after pregnancy [...], would be open to this programme [eHealth] because they have a double job with a household. They have no time to visit a pelvic floor physiotherapist and they are capable of understanding such an eHealth programme.” (GP6, female, ≤ 45 y).*

Older women were thought to have less benefit from PFMT compared with younger women. GPs admitted that they prescribed incontinence pads as a pragmatic option to them. Several GPs were not inclined to refer older women with co-morbidity for surgery because of risks of complications. EHealth was not an appropriate intervention for older women, according to several GPs. GPs thought they had no computer, or they associated older women with computer illiterates. One GP opposed the importance of chronological age and considered the biological age as far more important.

*“I think mostly younger people would be helped by eHealth. However, urinary incontinence in older people is more common, and a number of these people do not even know how to start a computer.” (GP12, male, > 45 y).*

*“If people seem to understand eHealth, then age does not matter to me. [...] I am against ageism.” (GP11, female, > 45 y).*

## Discussion

This study showed that GPs preferred PFMT, mostly by a physiotherapist, in their routine practice for SUI. EHealth with PFMT was welcomed by GPs as a new tool that they would recommend to their patients. For patients with SUI approachability and flexibility were considered as major advantages which improves access to care. GPs also mentioned that eHealth as a stand-alone intervention has its disadvantages, namely the lack of personal feedback and the risk that patients lose motivation due to absence of someone who supports them. Therefore, most GPs believed that personal support during an eHealth intervention would increase its effectiveness, but they were inconclusive about how and by whom support should be provided. The recommendation of eHealth depended on factors such as a patient’s motivation and preference, as well as her age.

The perceived advantages of eHealth in our study correspond with previous studies on eHealth interventions for other health problems. GPs consider approachability and flexibility to be major advantages for patients [26, 27], which improve access to care [28]. According to literature, GPs emphasise that the integration of eHealth within frequently used, evidence-based web pages eases access and is less time consuming [29–32].

GPs also mentioned disadvantages of eHealth as a stand-alone intervention which are related to the diagnostic procedure and to the monitoring of the intervention. They were concerned about the absence of a diagnostic procedure; women with a diagnosis other than SUI could incorrectly start with eHealth. Nonetheless, former studies suggest that women can accurately diagnose themselves through a questionnaire [33, 34]. Furthermore, GPs thought that the absence of support within eHealth could make the intervention less effective because women lose motivation to perform PFMT. However, studies on eHealth interventions for SUI have shown those interventions to be successful in improving SUI without face-to-face support [17, 18]. In addition, evidence is inconclusive in showing superiority of PFMT that is supervised by a physiotherapist, over home and non-supervised PFMT [35].

Some GPs preferred eHealth supported by a physiotherapist probably because GPs acknowledged their lack of affinity with pelvic floor problems, and they felt incapable of monitoring the strength of the pelvic floor muscle. Previous research on urinary incontinence in primary care confirmed these feelings of incapability, as well as lack of affinity [13]. EHealth has been shown to increase the feeling of being a more competent caregiver [21], which possibly explains why GPs in our study welcomed eHealth as a new tool.

Lastly, this study implicates that GPs might be prejudiced about age and treatment for women with SUI. GPs expected that young women would be more suitable for an eHealth intervention compared with older women, but a previous eHealth study for SUI showed that the probability of a successful outcome increases with age [36]. Caution is needed, however, as eHealth interventions for urinary incontinence are only studied in a relatively young population [17, 18]. Also within routine practice for SUI, GPs are prejudiced regarding a woman’s age as some GPs in this study thought that older women would not profit from PFMT. Nevertheless, a Cochrane review showed that PFMT is effective for women from different age groups [37]. GPs in this study were also not inclined to refer older women with co-morbidity to a specialist because of perceived risks of complications after surgery. However, evidence is inconclusive about differences in complication rates between older and younger women who underwent surgery for their incontinence [38]. These discrepancies between practice and evidence correspond with the findings from literature of well-known facts that women with urinary incontinence receive suboptimal treatment [10, 14].

Our study has some strength as it is the first to our best knowledge regarding attitudes of GPs about eHealth for urinary incontinence. As eHealth is a broad concept varying from telecommunication to web portals, it is difficult to compare different eHealth studies. Another strength is the inclusion of GPs with a variety in demographic characteristics. We included relatively young GPs whose attitudes are of special interest because these GPs are going to be confronted with eHealth in their future career. Our study also shows the limitation that the findings are not reflective for the whole profession of GPs because a qualitative study is by nature not generalisable to the wider population. Furthermore, we could not prevent response bias as we questioned GPs on a conceptual eHealth intervention [18], although we explained that it was based on the one used in the Swedish study.

The GPs in our study express reluctance with using eHealth as stand-alone intervention which shows that they are either not familiar with the current evidence on the topic, or that they found that the studies are not convincing. The GPs in our study also mentioned that before they would use or recommend eHealth they need to be sure that it is evidence-based. Trust in eHealth could increase with training that make GPs familiar with the existing evidence on potential future eHealth therapy [18, 28]. Furthermore, training is needed to improve current incontinence care, for example by addressing common misunderstandings among GPs such as the effectiveness of regular PFMT for different age groups. Before eHealth can operate as a stand-alone therapy, also research on an eHealth intervention in Dutch women with SUI might be necessary for these Dutch GPs to gain trust in its self-reliant aspects of eHealth. This process of making sense of an innovation is an important step for the implementation of an eHealth intervention for SUI, which is commonly overlooked within implementation studies [22]. The findings of this study are important for the design of the intervention. GPs were concerned that women for which eHealth is not satisfying might become discouraged to seek help. These concerns could be covered by providing information about when to consult a GP, within the eHealth application. Also, sending reminders throughout the course of the intervention will increase adherence to the training.

## Conclusions

EHealth based on PFMT is a welcome new tool that fits into the GPs’ practice for patients with SUI. Because of its approachability and flexibility, it could provide care to a broader group of patients who would otherwise not seek help. EHealth that functions as a stand-alone therapy needs trust from GPs, due to the perceived shortcoming of the absence of person support. GPs also thought that eHealth was no cure-all therapy, especially not for the elderly. Training should inform GPs about these new treatment possibilities for SUI and should focus on common misunderstandings about regular care for these women. Also more evidence or awareness is needed to convince the GPs on the added value of eHealth.

## Additional files

Additional file 1: Appendix 1 Interview guide. (DOCX 13 kb)

Additional file 2: Appendix 2 COREQ (DOCX 14 kb)

## Abbreviations

GP: General Practitioner
PFMT: Pelvic Floor Muscle Training
SUI: Stress Urinary Incontinence
