# Supplementary material for: Mixed feelings: general practitioners’ attitudes towards eHealth for stress urinary incontinence - a qualitative study
Source: BMC Fam Pract. 2019 Jan 26;20:21. doi: 10.1186/s12875-019-0907-x (PMC6347743; doi:10.1186/s12875-019-0907-x)
Supplement: Supplementary file 1 — Appendix 1 Interview guide. (DOCX 13 kb) [file 12875_2019_907_MOESM1_ESM.docx]

**Appendix 1 – Interview guide**

| **Topics** | **Example questions** |
| --- | --- |
| Routine practice for SUI | *Which treatment option for SUI do you prefer?*  *Which patient characteristics influence your decision-making for treatment of SUI?* |
| Experiences with eHealth in general | *What are your experiences with eHealth in general?* |
| Attitudes towards eHealth for SUI | *What is your opinion about eHealth for SUI, and what are pros and cons?*  *Which categories of patients would (not) benefit from eHealth for SUI?* |
| Personal support and eHealth for SUI | *What do you think of personal support during an eHealth-based therapy for SUI?* |
| Application of eHealth for SUI | *Would you apply an eHealth intervention for SUI into practice?*  *How would you apply eHealth for SUI into practice?* |
