# Supplementary material for: Mixed feelings: general practitioners’ attitudes towards eHealth for stress urinary incontinence - a qualitative study
Source: BMC Fam Pract. 2019 Jan 26;20:21. doi: 10.1186/s12875-019-0907-x (PMC6347743; doi:10.1186/s12875-019-0907-x)
Supplement: Supplementary file 2 — Appendix 2 COREQ (DOCX 14 kb) [file 12875_2019_907_MOESM2_ESM.docx]

**Appendix 2: COREQ guidelines**

**Domain 1: Personal characteristics**

1. Interviewer/facilitator: *CB interviewed all general practitioners*

2. Credentials:

*LF (Lotte Firet), PhD, general practitioner in training*

*CB (Chrissy de Bree), MSc*

*CV (Carmen Verhoeks), MSc*

*DT (Doreth Teunissen), PhD, MD*

*AL (Antoine Lagro-Janssen), Em. Prof, MD*

3. Occupation: occupation of CB: *medical student*

4. Gender: *all researchers (=authors) were female*

5. Experience and training: *The interviewer was trained in qualitative interviewing by the supervisors (DT and AL).*

6. Relationship established: *There was no relationship established prior to study commencement*.

7. Participant knowledge of the interviewer. What did the participants know about the researcher*? The interviewer told the participants about the goal of the study and that she was part of a project about eHealth for women with stress urinary incontinence.*

8. Interviewer characteristics: What characteristics were reported about the interviewer/facilitator?
*See 2, 4 an 7.*

**Domain 2: study design**

Theoretical framework

9. Methodological orientation and Theory: *We used a grounded theory approach in which we conducted semi-structured interviews that led to themes that describe the topic.*Participant selection

10. Sampling. *We selected the participants through purposive sampling.*

11. Method of approach. *Participants were recruited by ten general practitioners who are member of the expert group on urogynaecological diseases (ugynHAG). Each of these GPs approached two GP from their region who did not belong to the ugynHAG group. When a GP wanted to participate the GP from the ugynHAG group passed the contact details to the researcher. The researcher approached the GPs by e-mail.*

12. Sample size. *13 participants*

13. Non-participation. *7 GPs refused to participate because of practical aspects such as limited time.*Setting

14. Setting of data collection. *Interviews took place either face-to-face at the GP’s practice or through telephone, based on the GP’s preference.*

15. Presence of non-participants. *There was no other person present.*

16. Description of sample. *See table 1. Nine participants were female, eight were younger than 45 years old and most participants had their practice in an urban area.*

Data collection

17. Interview guide. *The interview guide was based on literature and on the expertise of the supervising committee (See appendix 1). We performed semi-structured interviews. Before the interviews were carried out, the interview guide was pilot tested twice.*

18. Repeat interviews. *Repeat interviews were not carried out.*

19. Audio/visual recording. *We made use of an audio recorder during the interviews.*

20. Field notes. *No field notes were made.*

21. Duration. *Interviews lasted 30 minutes on average.*

22. Data saturation. *Saturation was achieved after 11 interviews. The last two interviews have been conducted because the appointments for interviews were already set. These interviews revealed no new finding. Data saturation has been discussed.*

23. Transcripts returned. *Transcripts were not returned to participants.*

**Domain 3: analysis and findings**

Data analysis

24. Number of data coders. How many data coders coded the data? *After every three interviews two researchers independently coded the transcripts.*25. Description of the coding tree. *We did not provide a coding tree. Code list is available from the corresponding author on reasonable request.*

26. Derivation of themes. *Themes derived from the data.*

27. Software. *Atlas.ti version 7.1.5 was used.*

28. Participant checking. *Member checking was not perfomed.*

Reporting

29. Quotations presented. *Yes, quotations are displayed accompanied by identifier number, sex and age category.*

30. Data and findings consistent. *There was consistency between the data presented and the findings.*

31. Clarity of major themes. *We believe major themes are clearly presented.*

32. Clarity of minor themes. *In cases of inconsistencies within themes we provided nuance within the major themes. For example: “many GPs perceived eHealth as financially attractive […]. One GP warned against eHealth being used as an economic replacement for any other treatment.”* (page 8.
